# Locomotion and cadence detection using a single trunk-fixed accelerometer: validity for children with cerebral palsy in daily life-like conditions

**DOI:** 10.1186/s12984-019-0494-z

**Published:** 2019-02-04

**Authors:** Anisoara Paraschiv-Ionescu, Christopher Newman, Lena Carcreff, Corinna N. Gerber, Stephane Armand, Kamiar Aminian

**Affiliations:** 1 Laboratory of Movement Analysis and Measurement, Ecole Polytechnique Fédérale de Lausanne (EPFL), Station 9, CH-1015 Lausanne, Switzerland; 2 Paediatric Neurology and Neurorehabilitation Unit, Department of Pediatrics, Lausanne University Hospital, Lausanne, Switzerland; 3 Laboratory of Kinesiology Willy Taillard, Geneva University Hospitals and University of Geneva, Geneva, Switzerland

**Keywords:** Cerebral palsy, Atypical gait, Step detection, Accelerometer, Validation

## Abstract

**Background:**

Physical therapy interventions for ambulatory youth with cerebral palsy (CP) often focus on activity-based strategies to promote functional mobility and participation in physical activity. The use of activity monitors validated for this population could help to design effective personalized interventions by providing reliable outcome measures. The objective of this study was to devise a single-sensor based algorithm for locomotion and cadence detection, robust to atypical gait patterns of children with CP in the real-life like monitoring conditions.

**Methods:**

Study included 15 children with CP, classified according to Gross Motor Function Classification System (GMFCS) between levels I and III, and 11 age-matched typically developing (TD). Six IMU devices were fixed on participant’s trunk (chest and low back/L5), thighs, and shanks. IMUs on trunk were independently used for development of algorithm, whereas the ensemble of devices on lower limbs were used as reference system. Data was collected according to a semi-structured protocol, and included typical daily-life activities performed indoor and outdoor.

The algorithm was based on detection of peaks associated to heel-strike events, identified from the norm of trunk acceleration signals, and included several processing stages such as peak enhancement and selection of the steps-related peaks using heuristic decision rules. Cadence was estimated using time- and frequency–domain approaches. Performance metrics were sensitivity, specificity, precision, error, intra-class correlation coefficient, and Bland-Altman analysis.

**Results:**

According to GMFCS, CP children were classified as GMFCS I (*n* = 7), GMFCS II (*n* = 3) and GMFCS III (*n* = 5). Mean values of sensitivity, specificity and precision for locomotion detection ranged between 0.93–0.98, 0.92–0.97 and 0.86–0.98 for TD, CP-GMFCS I and CP-GMFCS II-III groups, respectively.

Mean values of absolute error for cadence estimation (steps/min) were similar for both methods, and ranged between 0.51–0.88, 1.18–1.33 and 1.94–2.3 for TD, CP-GMFCS I and CP-GMFCS II-III groups, respectively. The standard deviation was higher in CP-GMFCS II-III group, the lower performances being explained by the high variability of atypical gait patterns.

**Conclusions:**

The algorithm demonstrated good performance when applied to a wide range of gait patterns, from normal to the pathological gait of highly affected children with CP using walking aids.

## Introduction

Cerebral palsy (CP), caused by damage to the motor control networks of the immature brain, is the major cause of long-term physical disability in children [1]. Although the initial brain injury remains static, many affected children have progressive movement and posture impairments due to progressive musculoskeletal pathology (muscle weakness, spasticity, and bone deformity). Treatment options include physiotherapy, orthoses, pharmacological interventions, orthopedic and neurosurgical interventions [2]. Physical therapy interventions for the ambulatory youth with CP often focus on activity-based strategies to promote functional mobility in daily-life contexts and participation in physical activity [3]. An important component of functional mobility in ambulatory subjects is locomotion activity in the context of everyday life. The use of activity monitors specifically validated for this population could help to design effective personalized interventions by providing reliable outcome measures. Step counting using body worn accelerometer device(s) is one of the most common methods used to derive mobility-related metrics, such as the total number of steps per day, and duration and cadence of locomotion periods. However, the robust estimation of these parameters in real-life conditions is challenging, given the influence of environment (e.g. surface type/slope/stairs, indoor vs outdoor etc.) and the variability in movement disorders, as for instance in children with CP.

A previous study [4] has demonstrated acceptable validity of accelerometry (thigh-attached activPAL™ system) in young people with CP classified in level I (i.e., less affected) according to Gross Motor Function Classification System (GMFCS) [5]. However, further studies including subjects with CP-GMFCS levels I to III have indicated decreased step detection performances for the most affected subjects [6] [7]. The validity of other body worn activity monitors (Activity Monitoring Pad, consisting of a combination of inertial sensors, attached to the right lower leg above the ankle, and Minimod systems, consisting in a 3D accelerometer worn on the lower back) was evaluated by Kuo et al. [8] on an extended sample including typically developing (TD) children and children with CP-GMFCS levels I-III. These systems were able to accurately measure the number of steps and the time spent walking for the less complex hemiplegic gait patterns. Recently, a more sophisticated system (Pediatric SmartShoe), including FSR sensors located on an insole and a 3D accelerometer mounted on the heel of the shoe, was validated on a sample of children with CP classified CP-GMFCS I-II [9]. This system showed good accuracy for activity classification (sitting, standing, walking) and estimation of various gait parameters.

The common feature of the abovementioned studies is that validation data was collected using a structured protocol in laboratory settings. There is evidence and consensus in the literature suggesting that structured or standardized laboratory-based protocol lacks ecological validity, because activities are not performed in a natural way and order, and thus cannot be used alone to validate spontaneous activity in real-life [10]. Systems/algorithms validated only in laboratory settings may have lower accuracy when applied on data collected in real-life or collected according to protocols that mimic real-life settings [11–13].

The objective of this study was therefore to develop and validate a single-sensor based algorithm for detection of duration and cadence of locomotion periods, robust to the various pathological gait patterns in CP, in a real-life like environment, and placement of the sensor on the lower back (L5) or chest. A simple configuration, based on a sensor fixed on the upper-body, could be a preferable solution for large clinical studies, designed to assess daily-life physical functioning over long periods of time.

## Methodology

### Data collection

#### Participants

The study included fifteen children/adolescents with CP and eleven age- and sex-matched TD controls. Participants of the CP group were recruited from the patients followed at the pediatric orthopedics unit of Geneva University Hospitals (HUG). Inclusion criteria were: aged between 8 and 20 years, diagnosis of CP, ability to walk in the community with or without mechanical walking aids, and with a GMFCS level between I and III. For the control group, TD children were recruited among collaborators’ or patients’ acquaintances. The exclusion criteria for both groups were those that precluded adequate participation in the measurement sessions (mental age < 8 years, attention deficit and other significant behavioral issues, severe visual disorder). All participants and their parents/guardians provided written consent, and the protocol was approved by the hospital’s institutional ethical committee (CCER-15-176).

#### Measurement protocol

Each participant was equipped with six synchronized IMU devices (Physilog4®, Gait Up, CH, https://gaitup.com/wp-content/uploads/Brochure_Datasheet_Physilog_RA_V2.6.pdf) fixed on the chest (sternum), lower back (L5), tights and shanks using a hypoallergenic adhesive film (Opsite Flexigrid, Smith&Nephew Medical, Hull, UK). Physilog4® is a standalone device (dimensions: 50 mm × 37 mm × 9.2 mm, weight: 19 g) including 3D accelerometer, 3D gyroscope, 3D magnetometer and barometer with adjustable ranges, battery, a memory unit, and microcontroller. The sampling frequency was set at 100 Hz. IMU devices on the chest and L5 were independently used for development and validation of algorithms (locomotion detection and cadence estimation), whereas the devices on the lower limbs were used as reference system. The IMUs on lower limbs were aligned to the mediolateral axis to measure rotations (angular velocity) in the sagittal plane. The magnetometer was disabled.

The measurements took place at Laboratory of Kinesiology Willy Taillard, Geneva University Hospitals and Switzerland. Once equipped with the IMU devices, each participant performed a sequence of activities inside the hospital and outdoor in a park close to the hospital. The entire measurement session, which was expected to take approximately two hours, included walking indoor at various speeds, running, sitting down and standing up, changing floors using up/down stairs, walking outdoor on different surfaces (e.g. grass, gravel) and slopes, and spending time in the play park area. These activities were suggested to the participant in a way that flexibility was given on how and how long to be performed. This semi-structured data collection protocol was recommended whereby the participant performs a series of activities in a lifelike scenario at their comfortable speed, with or without walking aids and in the manner they are used to in daily life situations. This type of data collection is recommended for algorithm development before validation in real-life conditions [10].

*Reference/ground truth data:* During the monitoring period a research assistant followed the participant to record the timing of each activity using a custom designed application on a tablet (Samsung galaxy tab. E). At the end of monitoring a log file was generated which was subsequently downloaded on the computer and processed to generate a vector of symbols corresponding to duration and timing of activities performed (synchronized and resampled to correspond to IMU data). This data was used as reference (ground truth) for the type of activity (locomotion/walking/running vs. non-locomotion) and the context of locomotion (level, up/down stairs). The reference values for the number of steps and cadence of detected walking periods were obtained from the pitch angular velocity signal of both shanks and using a validated gait analysis algorithm [14–16]. Based on this algorithm, the maxima in the pitch shank angular velocity signal (i.e., rotation in sagittal plane), was considered as the instant corresponding to mid-swing. In the case of abnormal gait (e.g. most affected children and/or those using walking aids) the shank angular velocity signals were distorted, therefore in order to highlight the maxima we applied supplementary filtering (DWT, coiff5, approximation level 5) before mid-swing detection using the method described in [15]. The mid-swing events merged from the right and left leg were associated with the actual steps.

### Trunk sensor algorithms

#### Step/locomotion detection

Step detection algorithms are generally based on detection of peaks associated to heel-strike events, identified from trunk acceleration signals (chest or lower back) in the vertical direction [17, 18], anteroposterior direction [19, 20], or from the 3D acceleration norm [21]. In this study, in order to be insensitive to sensor placement and orientation, the algorithm was devised using the 3D acceleration norm (*accN*), defined as:

1 $$ accN=\sqrt{acc_V^2+{acc}_{AP}^2+{acc}_{ML}^2} $$

where *acc*_*V*_*, acc*_*AP*_ and *acc*_*ML*_ are the components of acceleration in vertical, anteroposterior (AP) and mediolateral (ML) directions, respectively.

A prior observation showed that in TD children the acceleration signals were similar step-by-step, and the steps appeared clearly identifiable by determining the local extrema (minima/maxima). Conversely, these signals showed considerable difference in morphology and amplitude among subjects with gait impairment and individual-specific compensatory movement strategies like in children with CP. The difference of acceleration signals between chest and L5 was quantified using the attenuation coefficient, defined as [22]:

2 $$ AC=\left(1-\frac{RMS_{a, chest}}{RMS_{a,L5}}\right)\times 100\left(\%\right) $$

where *RMS*_*a,chest*_ and *RMS*_*a,L5*_ are the root mean square of acceleration signal computed for the chest and L5 sensor, respectively.

**Fig. 1:**
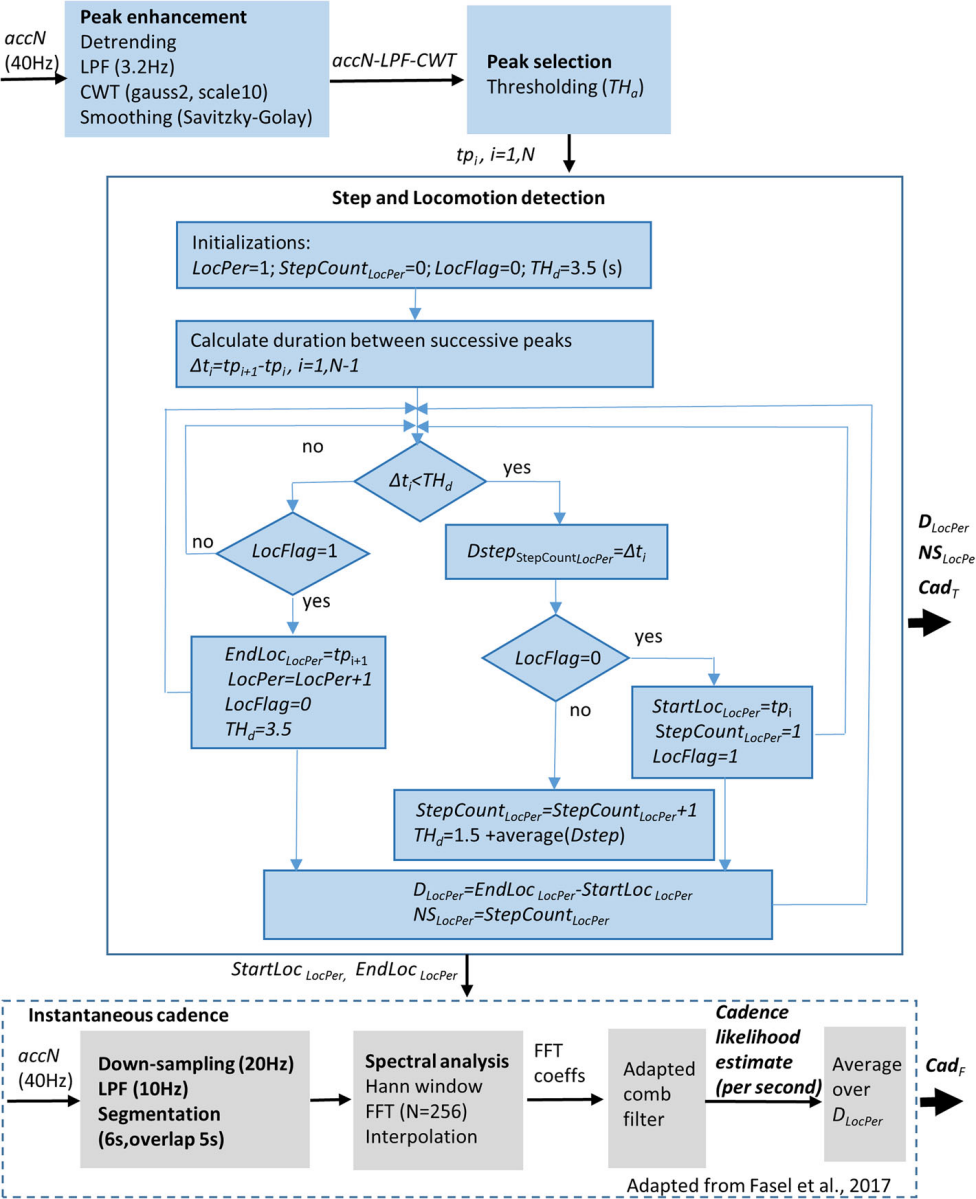
Flowchart of processing stages

In order to cope with the variability of the gait patterns and to reliably detect the locomotion steps when the sensor is located either on chest or L5, the algorithm included several processing stages as depicted in Fig. 1 and described below.

*Peak enhancement:* This first stage aimed to obtain a signal that contains steps-related information consistent among various gait patterns. The raw acceleration norm, *accN,* was first resampled at 40 Hz to correspond to a lower frequency adapted for long-term monitoring setups [23]. Subsequently, the signal was detrended and low-pass filtered (FIR filter, *n* = 120 coefficients, *Fc* ≈3.2 Hz) to give *accN-LPF.* The cutoff frequency *Fc* was chosen to allow detection of step cadence up to ≈ 195 steps/min (very fast running) while smoothing the signal by removing the high frequency noise. To precisely obtain zero-phase distortion, the filter was applied to the acceleration data twice, i.e., after filtering in the forward direction, the filtered sequence was reversed and run back through the filter (e.g. with filtfilt in Matlab). To further improve the signal-to-noise ratio and enhance steps-related peaks in the presence of artefact in impaired/atypical gait, we applied a smoothing and differentiation process using the continuous wavelet transform (*cwt*, scale 10, gauss2 wavelet in Matlab), [18, 24, 25], followed by a supplementary mild smoothing using a linear Savitzky-Golay filter (zero degree polynomial, smoothing frame length of 3 samples) to obtain the signal *accN-LPF-CWT.*

*Peak selection, step detection, and identification of locomotion periods:* From the processed acceleration signal *accN-LPF-CWT*, all the peaks with the amplitude located above a fixed threshold *TH*_*a*_ *= 0.1 (g)* were selected as potential heel-strike events, characterized by their occurrence time *tp*_*i*_, *i = 1,N*. A sensitivity analysis was conducted to choose the optimal value of *TH*_*a.*_ The next processing stage included detection of the actual steps and identification of the start/end of locomotion periods, as indicated in the flowchart in Fig. 1. The algorithm starts with initialization of several variables, such as the counter of locomotion periods (*LocPer*), the counter of steps belonging to the locomotion period (*StepCount*_*LocPer*_*),* a flag signaling the start/end of locomotion period (*LocFlag*), and a threshold used for comparison of duration between successive peaks (*TH*_*d.*_*)*_*.*_ Then, the duration between successive selected peaks, *Δt*_*i*_ *= tp*_*i + 1*_*- tp*_*i*_, *i = 1,N-1,* is compared with *TH*_*d*_ and if *Δt*_*i*_ *< TH*_*d*_, the step counter is incremented. At the beginning of each locomotion period the threshold is initialized with a fixed value of *TH*_*d*_ = 3.5 (s), and then it is updated at each iteration with the average value of duration of previous steps belonging to the current locomotion period, *TH*_*d*_ = 1.5 + average(*Dstep*) (s). The underlying idea is to adapt the threshold to the cadence/rhythm of the current locomotion period and thus improve the robustness of step detection algorithm in real world conditions and in various populations. The threshold values allow the detection of slow locomotion (minimal cadence around 35 steps/min), and avoid the interruption of faster locomotion periods when there are occasional undetected steps-related peaks between two consecutive selected peaks (e.g., during turning, gait asymmetry).

After detection of all locomotion periods only those containing at least four consecutive steps were retained as true locomotion and were used for further assessment. Each of these locomotion periods was characterized by the number of steps *N*_*steps*_ and its duration *D*_*loc period*_ (in minutes).

#### Cadence of locomotion periods

Two methods, using temporal and frequency-domain approaches, have been implemented to estimate the cadence of detected locomotion periods. The objective was to comparatively evaluate their performance, advantages and limitations.

In the temporal domain, cadence was calculated based on *N*_*steps*_ and *D*_*loc period*_ as:

*Cad*_*T*_ (steps/min) = *N*_*steps*_/*D*_*loc period*_

The estimation in frequency domain was based on the methodology developed for a wrist-worn accelerometer, described in [26]. As illustrated in Fig. 1, the main processing steps included low-pass filtering (*Fc* = 10 Hz) and segmentation of *accN* (down-sampled to 20 Hz) into 6 s windows (with 5 s overlap to obtain an estimate of cadence each second), spectral analysis using FFT (Hann window, *N* = 256), interpolation of FFT coefficients to increase frequency resolution, followed by cadence likelihood estimate using an adapted comb filter. The values of cadence estimated every second were averaged over the duration of the respective locomotion period to obtain *Cad*_*F*_.

#### Validation and statistical analysis

Similar to trunk algorithm, sequences of at least four consecutive steps (mid-swing events merged from left and right shank) were considered as locomotion periods, and were used as reference for duration (*D*_*ref*_) and cadence of locomotion periods (*Cad*_*ref*_).

The performance of the algorithm for detection of duration of locomotion periods was assessed in terms of sensitivity, specificity and precision. The value of these metrics can vary from 0 to 1, higher values indicating better performance. For cadence, the performance was assessed using absolute and relative error, intra-class correlation coefficient, ICC (A,1) [27], and Bland-Altman analysis. The significance level was set to *p* < 0.05.

## Results

Table 1 contains demographic and clinical data of study participants. There was no significant difference for age and gender between TD and CP groups. According to the GMFCS scale, children with CP were classified as GMFCS I (*n* = 7), GMFCS II (*n* = 3) and GMFCS III (*n* = 5). Those who were classified as GMFCS III used walking aids (rollators, crutches). Characteristics of the gait pattern and clinical profile of children with CP are also included in Table 1.

**Table 1:** Characteristics of study participants

	TD (*n* = 11)	CP-GMFCS I-III (*n* = 15)
Gender Number of girls in the group (%)	5 (45)	8 (55)
Age in years Mean(SD)	13.5(2.9)	12.8(3.1)
Gait pattern	normal	GMFCS I: close to normal (*n* = 3), drop foot (n = 3), stiff knee (*n* = 1) GMFCS II-III: crouch gait (*n* = 4), equinus and recurvatum knees/stiff knees (*n* = 3), jump knee(*n* = 1)
Clinical profile		GMFCS I: hemiplegia (*n* = 4), diplegia (*n* = 3) GMFCS II-III: diplegia (*n* = 4), trilegia (*n* = 2), tetraplegia (*n* = 1), hereditary spastic paraplegia (*n* = 1)

To evaluate how severity of CP and the atypical gait pattern affect the performance of algorithm, children with GMFCS II and GMFCS III were pooled together and the performance metrics were presented comparatively for three groups i.e., TD, CP-GMFCS I, and CP-GMFCS II-III.

**Fig. 2:**
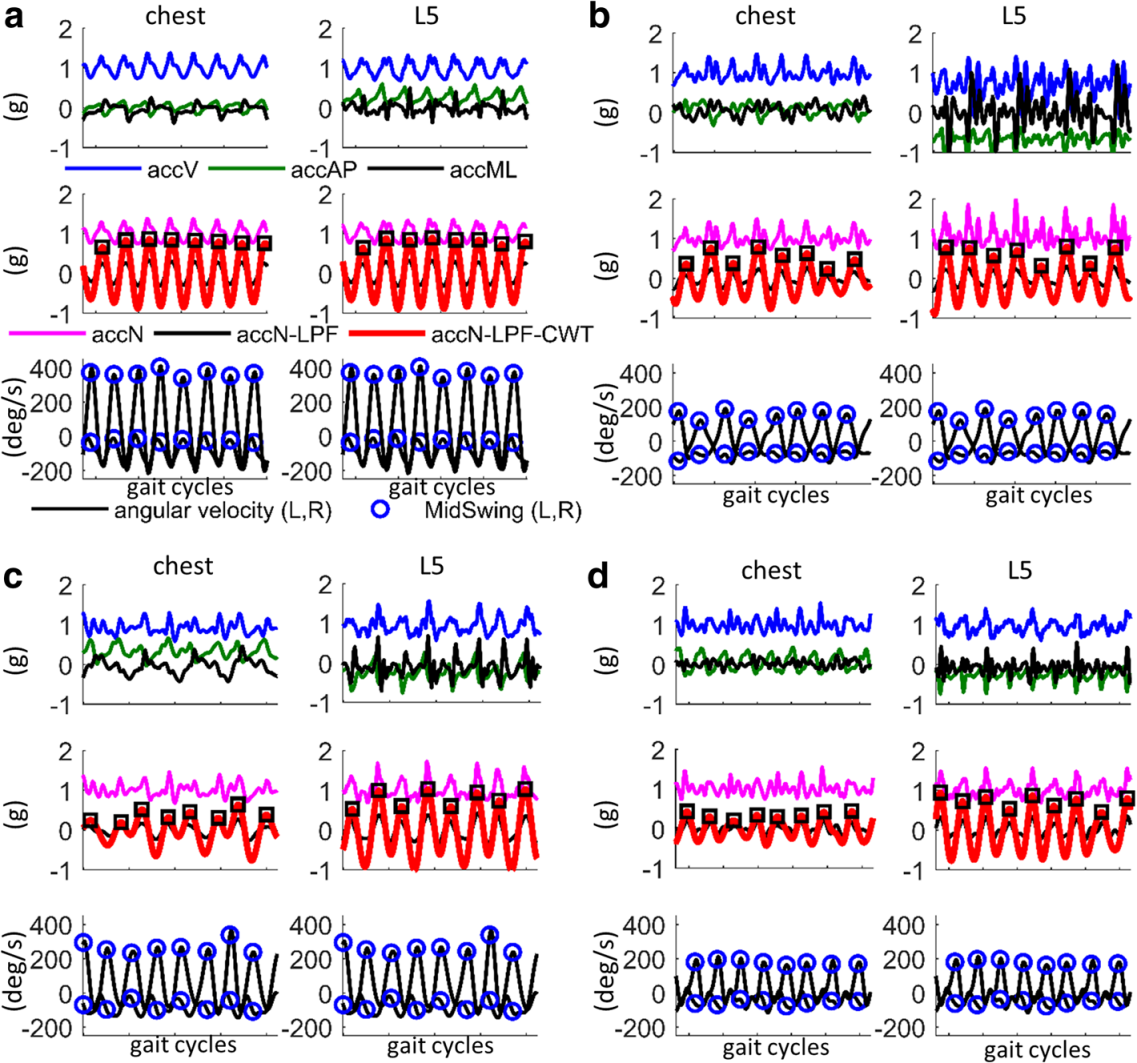
Acceleration signals recorded on chest and L5 for children with various gait patterns: **a**) TD child with normal gait, **b**) child with CP-GMFCS III, true equinus and recurvatum knees; **c**) child with CP-GMFCS III, apparent equinus (right side)/crouch (left side) with stiff knees; **d**) child with CP-GMFCS III, crouch gait. For each subject, the top panel illustrates the raw acceleration along the three axes, i.e., vertical (*accV*), anteroposterior (*accAP*) and mediolateral (*accML*). The middle panel shows the raw acceleration norm (*accN*, magenta color), after *detrending* and *LPF (accN-LPF*, black color), and after *continuous wavelet transform (accN-LPF-CWT,* red color*)*; steps are identified as the maxima corresponding to heel-strike events (black squares). The bottom panel shows the pitch angular velocity signals recorded on shanks; the reference steps (ground truth) were identified as the maxima corresponding to the mid swing temporal events (blue circles)

**Fig. 3:**
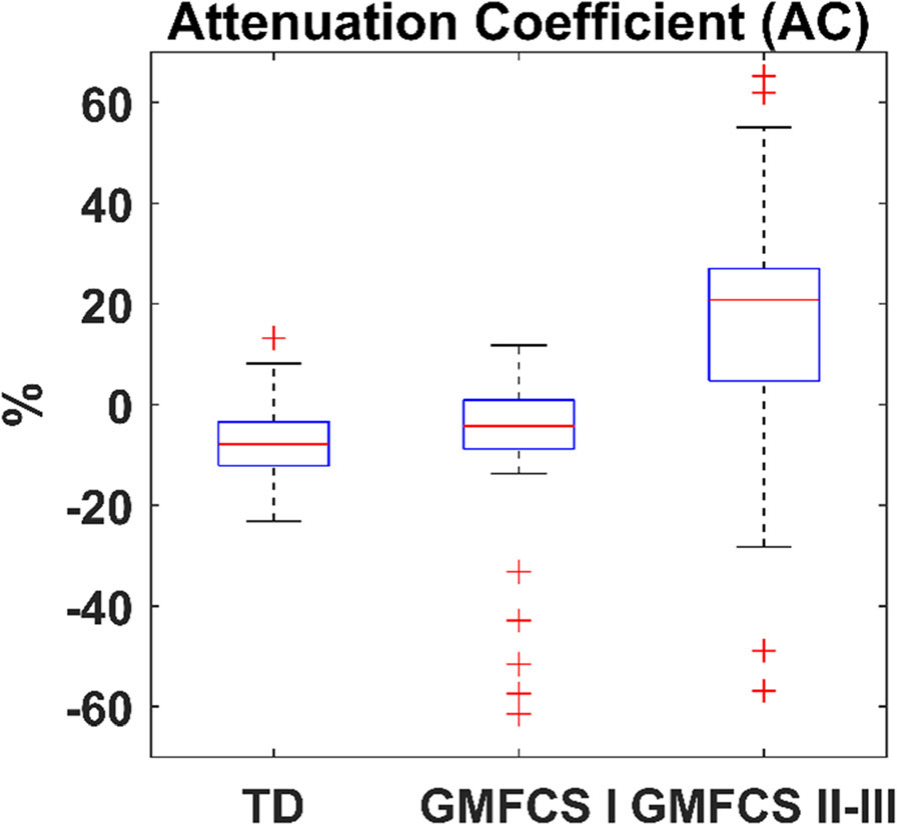
Attenuation coefficient illustrating a reduction of the acceleration from L5 to chest, especially for children with CP- GMFCS levels II and III

Figure 2 shows illustrative examples of acceleration signals recorded on chest and L5 during a few gait cycles, in three children with CP with atypical gait (Fig. 2b-d) compared to a TD child with normal gait (Fig. 2a). In addition to inter-subject variability, it can be observed that the patterns of acceleration signals differ also between chest and L5 locations, especially for highly affected children. The attenuation coefficient, *AC,* calculated for the processed acceleration signal (*accN-LPF-CWT*) showed a significant increase and a large variability for the group CP-GMFCS II-III, as compared to TD and CP-GMFCS I groups (Fig. 3).

Despite of these distorted signals our algorithm showed step detection performances similar for chest and L5 sensor, in agreement with the reference values obtained from the algorithm based on shank angular velocity signals.

### Locomotion periods

Mean and standard deviation (SD) of the performance metrics for locomotion detection using the IMU sensor fixed on chest or L5 are presented for each group in Table 2. For TD and CP-GMFCS I groups the performance metrics (*sensitivity*, *specificity* and *precision)* were relatively similar between chest and L5 sensor, with values ranging from 0.92 to 0.98. The minimal values were observed in CP-GMFCS II-III group where the chest sensor showed lower performance in term of *precision*, as compared to L5 (0.86 for chest compared to 0.93 for L5).

**Table 2:** Performance metrics for detection of locomotion periods as Mean(SD) for each group

	Sensitivity		Specificity		Precision	
	Chest	L5	Chest	L5	Chest	L5
TD	0.93 (0.01)	0.93 (0.01)	0.97 (0.01)	0.90 (0.01)	0.93 (0.02)	0.94 (0.02)
CP-GMFCS I	0.92 (0.01)	0.94 (0.01)	0.96 (0.01)	0.97 (0.01)	0.92 (0.03)	0.92 (0.03)
CP-GMFCS II-III	0.90 (0.06)	0.90 (0.08)	0.95 (0.02)	0.98 (0.01)	0.86 (0.07)	0.93 (0.03)

### Cadence

**Fig. 4:**
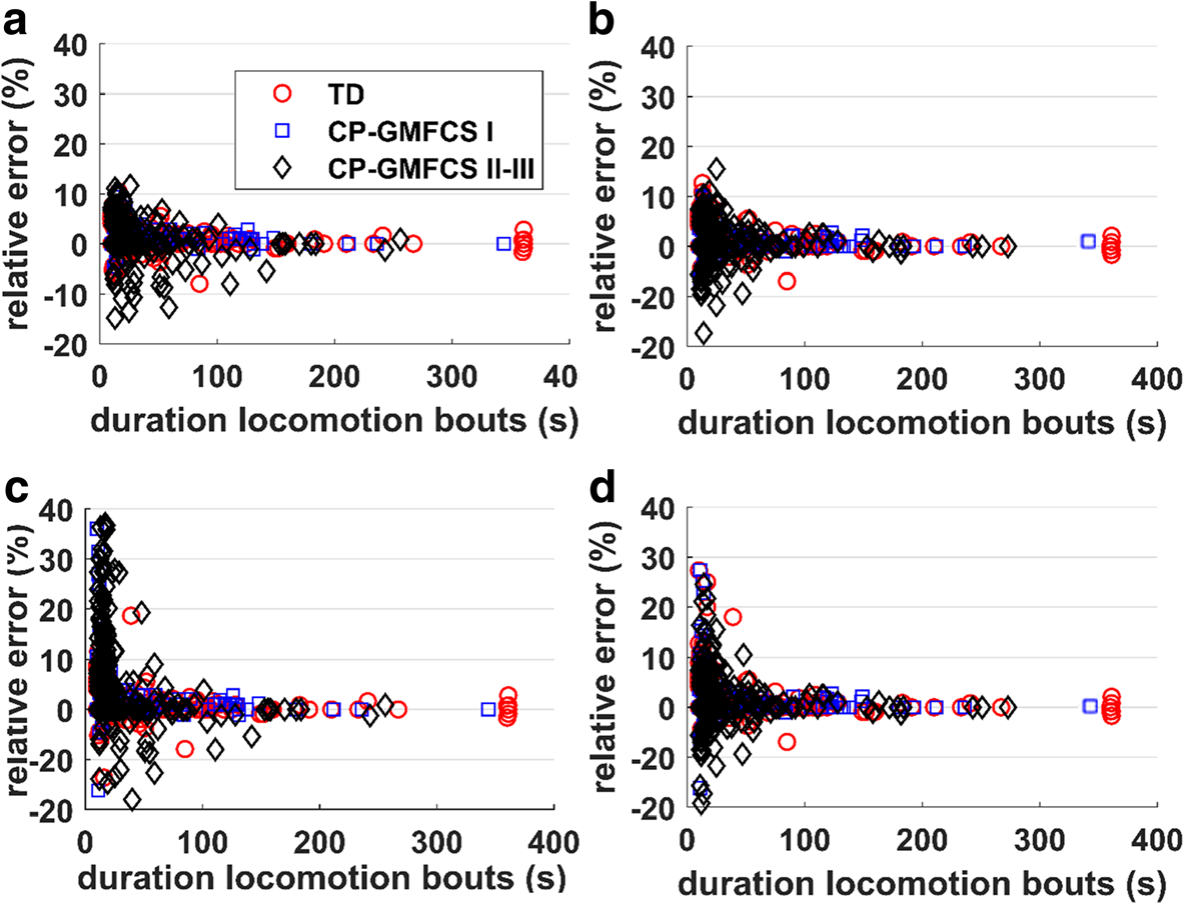
Variation of the relative error as a function of duration of locomotion periods: **a**), **b**) cadence measured in time domain from sensor on chest and L5, respectively; **c**), **d**) cadence estimated in time domain from sensor on chest and L5, respectively. The abrupt decrease of the error for locomotion periods longer than approximately 20 s, and the steadiness after, indicate that the longer periods, who are likely to correspond to purposeful locomotion, are more reliable for the assessment of the gait pattern in every-day life conditions

*Effect of duration of locomotion period*: The time domain approach provides a *measurement* of cadence based on the number of steps, whereas the frequency domain approach provides an *estimate* of cadence based on spectral analysis of acceleration signal segmented in windows of 6 s duration. When the duration of locomotion period is short and/or the gait pattern is unsteady (high variability), the error can be important. Figure 4 illustrates the variation of the relative error of *Cad*_*T*_ and *Cad*_*F*_ as a function of duration of locomotion periods. It can be observed that the error is higher for short periods, especially for frequency domain approach, due to lack of steady samples necessary to extract the spectral contents (Fig. 4c, d). Interestingly, an abrupt decrease of the error occurs for locomotion periods of approximately 20 s, and then becomes stable, a trend which is consistent for both, temporal and frequency domain, as well as sensor location. Given the difference between the two approaches for very short periods and guidelines from studies reported in literature, indicating that gait impairments/limitations appear more evident when looking at longer locomotion periods (i.e., purposeful walking) [28], the error analysis was conducted comparatively between the two approaches for locomotion periods lasting at least 20 s.

*Cadence errors for walking periods lasting minimum 20 s*: Tables 3 and 4 contain the errors for estimation of *Cad*_*T*_ and *Cad*_*F*_ respectively, for both sensor locations. The errors were low (mean absolute error less than 1.3 steps/min) and appeared quite similar for TD and CP-GMFCS I groups, when comparing chest and L5 locations, for time and frequency approaches. For CP-GMFCS II-III group the mean error was slightly higher (mean absolute error approx. 2 steps/min), but the standard deviation was high (up to approx. 9 steps/min), as a result of inhomogeneous results in this group due to the atypical and heterogeneous gait patterns.

**Table 3:** Performance metrics for cadence measured in time domain (*Cad*_*T*_), as Mean(SD) for each group

	Absolute Error (steps/min)		Relative Error (%)	
	Chest	L5	Chest	L5
TD	0.5 (1.9)	0.5 (1.9)	0.4 (2.0)	0.5 (2.0)
CP-GMFCS I	1.3 (1.4)	1.1 (1.3)	1.0 (1.0)	1.1 (1.0)
CP-GMFCS II-III	2.2 (7.4)	2.0 (6.8)	1.0 (1.0)	1.0 (7.0)

**Table 4:** Performance metrics for cadence estimated in frequency domain (*Cad*_*F*_*),* as Mean(SD) for each group

	Absolute Error (steps/min)		Relative Error (%)	
	Chest	L5	Chest	L5
TD	0.8 (3.5)	0.8 (3.4)	0.7 (2.0)	1.0 (5.0)
CP-GMFCS I	1.3 (1.4)	1.2 (1.4)	1.0 (1.0)	1.0 (1.0)
CP-GMFCS II-III	2.3 (8.7)	1.9 (7.2)	2.0 (9.0)	1.0 (6.0)

**Fig. 5:**
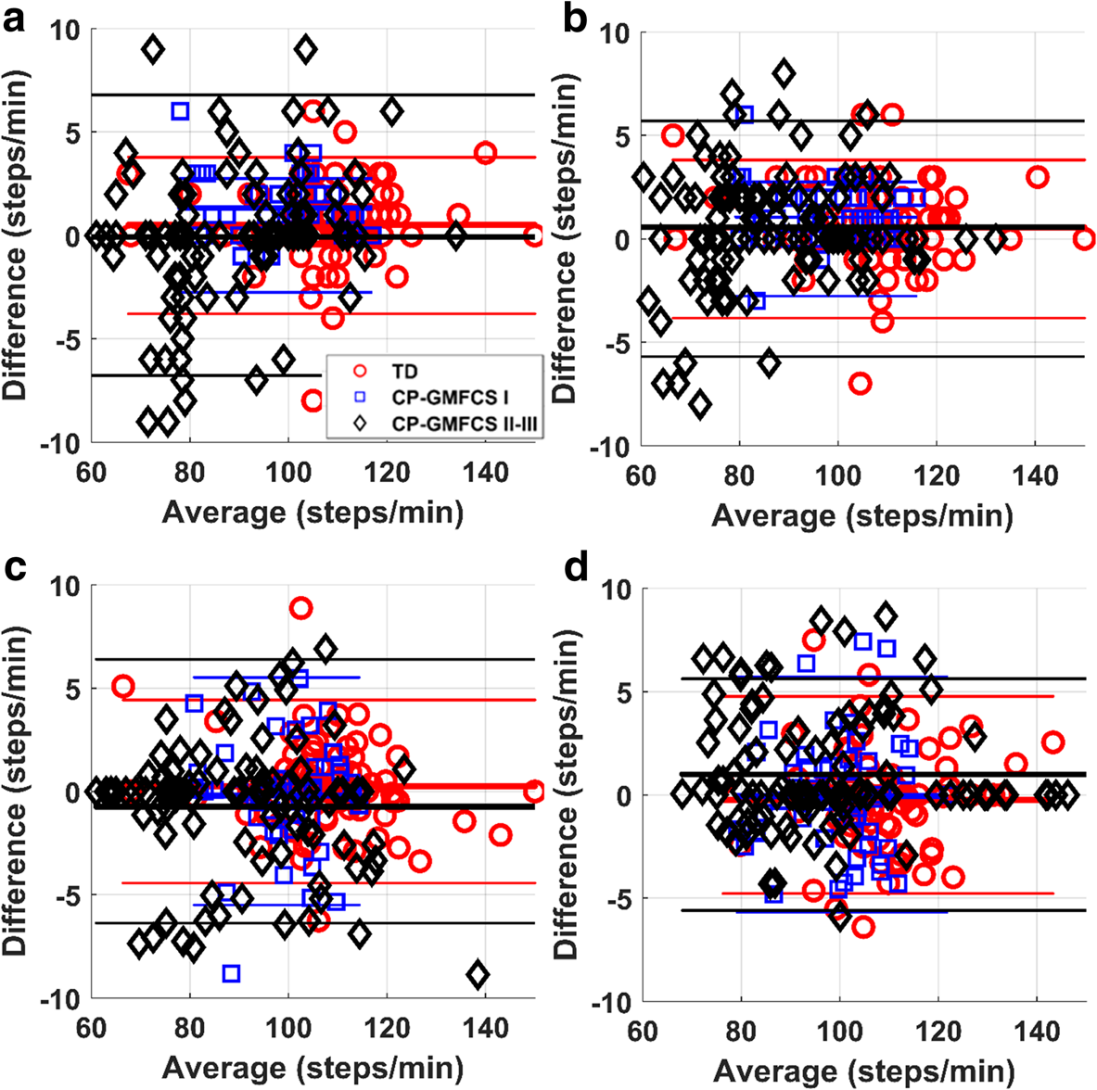
Bland-Altman plot for cadence: **a**), **b**) measurement in temporal domain using chest and L5 sensor, respectively; **c**), **d**) estimation in frequency domain using the sensor on chest and L5, respectively

Bland-Altman analysis (Fig. 5 a-d) revealed a small systematic error (bias) ranging from 0 to 1 step/min, across sensor locations, cadence estimation approaches, and groups of subjects. The wider limits of agreement (95% CI, or ± 1.96SD) was observed for CP-GMFCS II-III group, with values of approximately ±6 steps/min for the chest sensor and close to ±5 steps/min for L5 sensor. The narrowed limits for all groups were obtained for L5 sensor using the time-domain approach (Fig. 5b). The ICC(A,1) values were superior to 0.9.

**Fig. 6:**
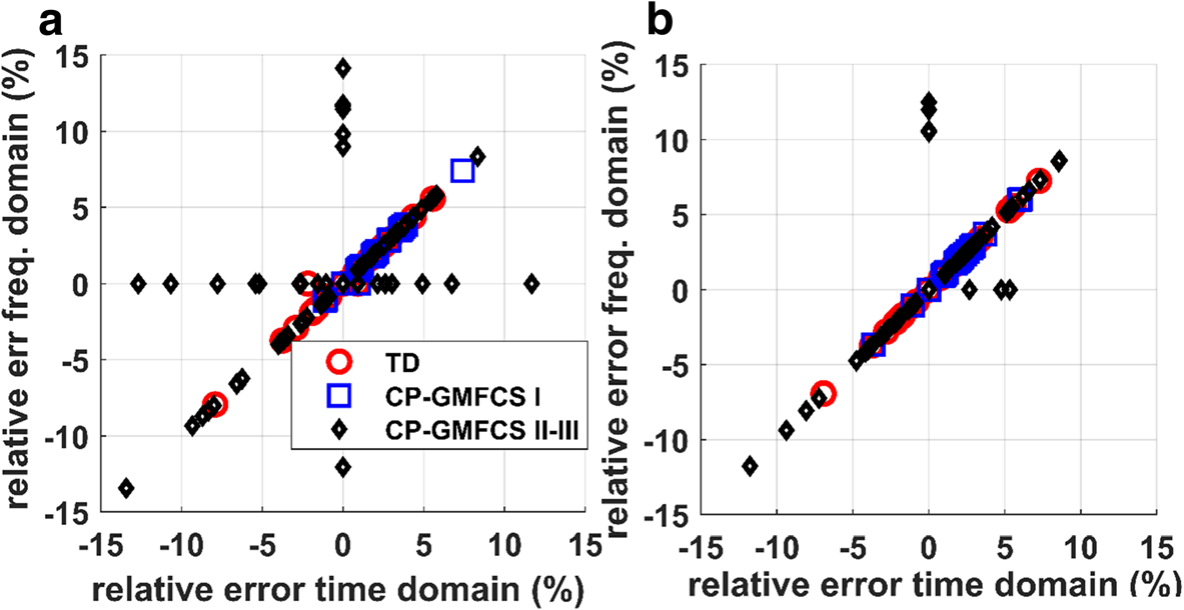
Relationship of the relative error for time and frequency domain methods: **a**) sensor on chest; **b**) sensor on L5

The scatterplots in Fig. 6 show the relationship between the relative error of *Cad*_*F*_ and *Cad*_*T*_. It was observed a linear association for both sensors, although there were a few more outliers for the chest sensor (e.g. error close to zero for *Cad*_*F*_ and variable over a wide range for *Cad*_*T*_, Fig. 6a), compared to L5 sensor (Fig. 6b).

## Discussion

Optimal sensor configuration for physical activity assessment in daily-life environments by reducing the number to a single adequate location is fundamental for clinical evaluation and subject adherence, particularly in individuals with physical impairments. The single IMU-based algorithm for step/locomotion detection and cadence estimation developed in this study demonstrated a good performance when applied to a wide range of gait patterns, from normal to the pathological gait of highly affected children with CP using walking aids.

The proposed algorithm is based on the norm of acceleration signal which has the advantage of being less sensitive to the orientation of the sensor with regard to the body segment. Actually, most of the trunk-based step detection algorithms use the acceleration signal in vertical or AP directions. Although the pattern of these signals contains more reliable information for step detection (as compared to ML direction), the algorithms using these signals necessitate the correction of sensor orientation using pre-defined functional calibration procedures [18, 20], an approach difficult to apply for real life monitoring, particularly in patients with movement disorders like children with CP. The inclusion of acceleration in ML direction for computation of the acceleration norm challenged the performances of the algorithm, because ML direction contained stride-related information (similar peaks in acceleration signal at every two steps instead of at each step) and artefacts arising from compensatory movement strategies.

Performances were relatively similar for chest and L5 sensor, despite the significant difference in acceleration signals, especially for CP-GMFCS II-III group (Figs. 2, 3). The significant reduction of the acceleration from L5 to chest (positive attenuation coefficient) in CP-GMFCS II-III group as compared to TD children confirms the results of previous studies [22]. Although the current version of the algorithm shows good performance, this could be potentially improved using more sophisticated approaches such as personalization by automatic setting of algorithm parameters. For example, it was observed that the peak enhancement stage significantly affects the step detection accuracy. The signal processing steps described in Fig. 1 were the optimal solution for the whole dataset; however it was observed that a more aggressive smoothing (e.g., cwt, scale 11, 12) of acceleration data recorded in patients with CP-GMFCS II-III improved algorithm accuracy for some of them. One possible solution for future developments could be a subject-specific adaptive filtering, based on ad-hoc characterization of signal features. The robust implementation and validation of this approach would however necessitate a large amount of data including a wide array of atypical gait patterns.

Time and frequency based domain approaches were proposed for cadence estimation. Each of these methods has specific advantages and limitations. As compared to the frequency based domain, the measurement in time domain is more accurate for short locomotion periods since it is based on peak detection; moreover, the identification of steps in time domain may allow detection of the temporal gait parameters [18, 20] and consequently a more detailed gait analysis. On the other hand, the measurement in frequency domain is more robust to outliers in acceleration signal and can provides an estimation of instantaneous cadence (e.g. every second) - a parameter useful to assess gait variability [26]. Although, on average, the performances were similar for the two approaches (Tables 3, 4, Fig. 6), the frequency-domain method appeared to slightly outperform the time-domain method for the chest sensor (the few cases where the error for *Cad*_*F*_ is close to zero, while the error for *Cad*_*T,*_ varies over a wide range).

Similar to previous studies [26, 29, 30], our results showed that the error for cadence detection decreases for longer locomotion periods. The increased error for shorter periods can be explained by undetected steps at the beginning and end of locomotion period, curved locomotion paths, slow walking or insufficient steady samples for spectral analysis when using frequency domain approach. However, in real-life conditions the brief periods usually correspond to short distance locomotion (e.g. less than 20 m) in constrained environments (e.g., stepping in home or indoor environment), therefore the interpretation of their cadence as the locomotion/functional ability of the subject is not straightforward [28].

### Strengths and limitations

The strengths of this study included the development and validation of the algorithm on an array of gait patterns, using data collected in a real-life like monitoring setting using and IMU device located either on chest or L5. This is an important aspect given the heterogeneity of disease severity and gait abnormality in various clinical populations, including individuals with CP.

However, a number of limitations must also be acknowledged. Although the overall sample size and data collected were adequate to ensure the statistical power of the performance metrics, it was insufficient to allow robust assessment for the subgroups of participants, especially for CP-GMFCS II-III. Within this group, the performances for both, locomotion and cadence detection were lower and highly variable between participants. Given the clinical importance of this group, for both medical assessment and intervention, further work would be necessary to improve the algorithm and examine the robustness on a larger sample of youths with severe CP. One of the main issue with this population when data is collected using real-life like protocols, is the availability of the ground truth for step number (cadence). For highly affected individuals using walking aids, step detection is difficult even with IMU devices on lower limbs. It is clear that inaccuracy in the reference data negatively affects the validation procedure. Therefore, further work is also necessary to improve the performances of gait/step detection algorithms using IMU devices on lower limbs [16]. This is particularly important since lower-limb IMUs is the most appropriate reference system for next validation phases, based on long-term recorded data in the actual everyday life context of the individuals [10].

It is worth mentioning that the signal processing for peak enhancement (Figs. 1, 2) allows detection of the most prominent steps-related peaks, associated to specific temporal events, i.e., heel-strike for trunk acceleration and mid-swing for shank pitch angular velocity. This smoothing procedure may lead to loss of information related to additional temporal parameters, therefore may appear less appropriate for detection of step duration.

Finally, the error for cadence estimation using both, time and frequency-domain methods, was low and stable, for locomotion episodes lasting for minimum 20 s duration. Although studies conducted on different clinical populations indicated that in order to assess gait/functional ability it is more appropriate to consider the long locomotion periods because are supposed to correspond to purposeful and more physically demanding tasks [28], these periods may represent only a low percent of locomotion in everyday life context [31], especially in individuals with severe gait impairments. The proposed algorithm might therefore be improved to decrease the error for the short locomotion periods.

## Conclusion

In this study we developed and validated a single-sensor based algorithm for locomotion and cadence detection that showed good performances for various gait patterns. Validation of the algorithms on heterogeneous populations is particularly important for subsequent cross-sectional and/or interventional studies when outcome measures are derived from locomotion features. Indeed the target goal of many intervention programs is defined according to normative values obtained from age−/gender-matched healthy subjects. Therefore, a reliable comparison of the outcome measures between subjects/groups requires monitoring and analysis in similar conditions using a unique robust algorithm.

## Abbreviations

3D: Three-dimensional
*accN*: acceleration norm
*accN-LPF*: acceleration norm after low-pass filtering
*accN-LPF-CWT*: acceleration norm after low-pass filtering and continuous wavelet transform
AP: anteroposterior
CP: cerebral palsy
CWT: continuous wavelet transform
DWT: Discrete Wavelet Transform
FSR: Force Sensitive Resistor
GMFCS: Gross Motor Function Classification System
ICC: Intra-class correlation coefficient
IMU: Inertial Measurement Unit
ML: mediolateral
RMS: root mean square
SD: standard deviation
TD: typically developing
