# Correction to: Locomotion and cadence detection using a single trunk-fixed accelerometer: validity for children with cerebral palsy in daily life-like conditions

**DOI:** 10.1186/s12984-019-0498-8

**Published:** 2019-02-12

**Authors:** Anisoara Paraschiv-Ionescu, Christopher J. Newman, Lena Carcreff, Corinna N. Gerber, Stephane Armand, Kamiar Aminian

**Affiliations:** 1 Laboratory of Movement Analysis and Measurement, Ecole Polytechnique Fédérale de Lausanne (EPFL), Station 9, CH-1015 Lausanne, Switzerland; 2 Paediatric Neurology and Neurorehabilitation Unit, Department of Pediatrics, Lausanne University Hospital, Lausanne, Switzerland; 3 Laboratory of Kinesiology Willy Taillard, Geneva University Hospitals and University of Geneva, Geneva, Switzerland

**Correction to: J Neuroeng Rehabil**

**https://doi.org/10.1186/s12984-019-0494-z**

The original article [1] contained a minor error whereby the middle initial of Christopher J. Newman’s name was mistakenly omitted. This has now been rectified.
